# A Comprehensive Review on the Anti-Cancer Effects of Oleuropein

**DOI:** 10.3390/life12081140

**Published:** 2022-07-28

**Authors:** Sabreen Rishmawi, Fatma Haddad, Ghadeer Dokmak, Rafik Karaman

**Affiliations:** 1 Pharmaceutical Sciences Department, Faculty of Pharmacy, Al-Quds University, Abu Dis, Jerusalem 9103401, Palestine; sabreen.rishmawi2@students.alquds.edu (S.R.); iamfromhebron@hotmail.com (F.H.); ghadeer_88@live.com (G.D.); 2 Faculty of Life Sciences, University of Bradford, Bradford BD7 1DP, UK; 3 Department of Sciences, University of Basilicata, 85100 Potenza, Italy

**Keywords:** Oleuropein, olive oil, cancer, *Olea europaea*, antioxidant, antiproliferative, angiogenesis, apoptosis

## Abstract

In Mediterranean cuisine and culture, olive oil and olive fruits play a significant role. Many people believe that those who consume olive oil and its fruit live longer and have a decreased risk of illness. Olive leaves were used to treat a range of diseases in ancient times, including malaria fever and lower earaches. Although it was not understood at the time what key components were responsible for these effects because they had not yet been discovered, Oleuropein is now recognized as one of the primary elements in immature olive fruits and leaves. Later research was carried out to determine the effects of this molecule, and it was determined that it functions as an antioxidant. Oleuropein consumption has aided in cancer treatment over the years, and this was assumed to be owing to its antioxidant properties. Oleuropein’s effects on cancer, however, go beyond that; it is now known that Oleuropein functions as both an anti-proliferative and an apoptotic promoter in many cancer cells. The kinetics and dosages of Oleuropein and the mechanisms behind its involvement and effects in cancer are explored in this review. Finally, the effects of Oleuropein in combination with anticancer medicines are investigated.

## 1. Introduction

**Figure 1:**
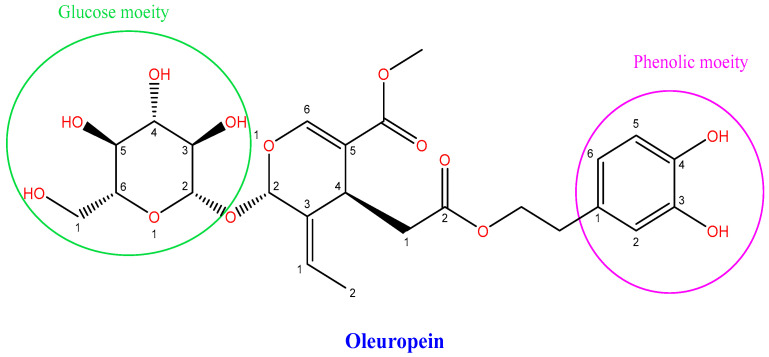
A chemical structure of Oleuropein [7].

Longevity and lower morbidity and mortality have long been associated with olive oil use in the Mediterranean diet [1]. Olive leaves have been used to treat malaria fever since ancient times [2], and numerous studies have shown that olive oil and olive leaves can enhance health by reducing cardiovascular and neurological illnesses [3]. Oleuropein (Ole) is the principal phenolic chemical found in all sections of the olive tree *Olea europaea* L., and their health advantages are described below [4], as it is found in all parts of the tree [5], particularly in raw olive fruit and leaves [1]. Ole (Figure 1) is an ester of oleanolic acid and hydroxytyrosol (HT) [1,3], which was discovered in 1908 [2,3]. Ole aglycone is generated when Ole is hydrolyzed during the mechanical extraction of green olives [1,5], and it has a bitter taste [1,2]. Ole is broken down into HT, which is found in abundance in processed olive oil and fruit [3]. Ole can be degraded chemically or enzymatically [3]. Many factors influence the amount of Ole in olive trees, including cultivar and production area [1], as well as soil moisture content, pollutants, and atmospheric conditions [2].

Many studies have shown that olive oil consumption reduces the incidence of cancer of any kind, particularly breast and digestive system tumors [5]. Many recent studies have suggested that Ole may play a role in cancer [1]; this impact is thought to be owing to Ole’s significant antioxidant qualities [2]. The effects of Ole on cancer cells are influenced by Ole concentration, exposure time, and cancer cell type, which explain the various processes by which Ole works [6].

## 2. Pharmacokinetics of Ole

### 2.1. Dose of Ole

Ole or olive leaf extract is available in a variety of formats as a dry extract [8]. Some studies suggest that 7.5 g of Ole for a 70 kg human may have an anti-tumor effect by decreasing mitosis and by increasing apoptosis [9,10], but this high dose may be impossible to achieve because the commercially available supplement of the olive leave extract contains approximately 20 mg−50 mg of Ole [11,12,13]. Ole at high levels, such as 50 mg/kg, is non-toxic [14].

### 2.2. Metabolism of Ole

Ole is absorbed in the small intestine and colon of animals and humans [15], undergoes high first-pass metabolism, forms sulfate and glucuronide conjugations [16], is degraded to HT in the large intestine, binds circulating human lipoproteins, and is excreted in urine as glucuronide conjugates [15]. The free form of Ole cannot be detected since it is quickly transformed into HT [16]. Because ole is resistant to stomach acidity, it does not undergo hydrolysis in the stomach [17].

It is poorly absorbed in the isolated perfused rat gut, according to Ole’s in situ test, whereas HT absorption is greater [18]. It is worth noting that, according to one study, Ole is not the primary source of HT [13]. In an experiment on freely moving rats, Ole showed the slowest absorption profile compared with HT, with just a little amount of Ole detected in plasma and urine following an oral dose (300 mg/kg; 555.02 µmol/kg) [18]. Ole was found in the plasma 5 min after receiving IV therapy (10 mg/kg; 18.50 µmol/kg) and was not found in urine; however, it was found in bile [18]. Ole metabolism is mediated by a number of metabolic processes, including de-glycosylation, hydrolysis, oxygenation, and methylation, according to that study, which explains why Ole was detected in bile [19].

We found some published human data on Ole, one of which looked at the excretion and metabolism of Ole after acute and chronic ingestion of olive leaf supplement [13,20]. The supplement came in the form of a pill with 20 mg of Ole and a liquid with 22 mg/5 mL of Ole. Three capsules or 15 mL were taken three times daily in the acute consumption [13]. This study discovered that phase II metabolites were detected in urine samples after chronic intake but not after acute ingestion, when five different Ole aglycone metabolites but no HT in any sample were detected [13]. Ole in its glycosylated form is absorbed through the small intestine wall [21], perhaps due to its polarity [16] or maybe through the use of a glucose transporter [21], according to the findings. It functions as a glucose carrier by facilitated and active diffusion [16,20]. In contrast to a prior rat study, which found that bacteria in the large intestine converts Ole into HT [1], in vivo observations have demonstrated that this is not the case [21].

Five male and five female middle-aged human volunteers were given two single doses of Ole, one week apart, with different doses of Ole, lower (51.1 mg) and higher (76.6 mg), so that at the end of the trial, each subject received the same quantity of Ole. There were no side effects or impaired liver function found in the trial [22]. The maximal concentration of Ole plasma in liquid form was six times greater than in capsule form. Gender, enzymatic makeup, and preparation form are all aspects that influence Ole metabolism and absorption, according to the study in [23]. There was a variation in Ole metabolism between post-menopausal and pre-menopausal women, according to another study [24]. To better understand pharmacokinetics, more human studies are needed to discover the various factors influencing Ole metabolism and absorption.

This review paper seeks to educate readers on anticancer activity, the mechanisms underlying it, and its potential anticancer benefits when combined with anticancer drugs.

## 3. Role of Ole in Cancer

### 3.1. Anti-Proliferative Effects of Ole

Researchers have discovered that, following Ole treatment at various IC50 doses (0.5× IC50, IC50, and 2× IC50), the proliferation of in vitro MCF-7 breast cancer cells diminishes in a time-dependent way [25]. Ole’s anti-proliferative action has been established in numerous research using MCF-7 cell lines (Table 1). Researchers have noted that the benefits of Ole may be preventative rather than therapeutic [26]. In vivo studies on mice that were subcutaneously injected with MCF-7 and given 125 mg/kg of Ole in their food revealed that it suppresses peri pulmonary and parenchymal lung metastases [27].

**Figure 2:**
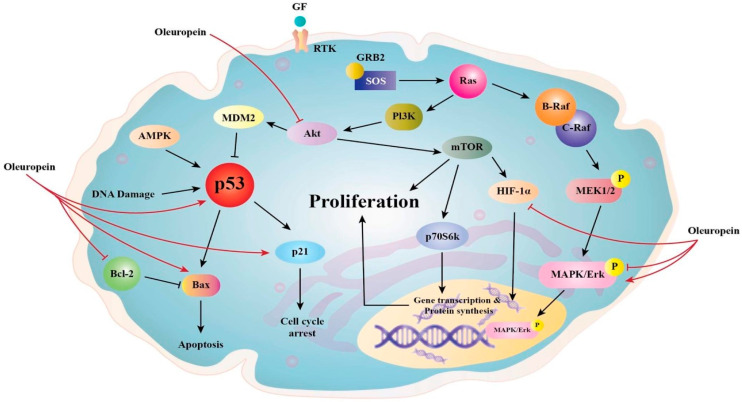
Proposed anti-proliferative mechanisms of Ole.

Ole (100 µM) was found to suppress the nuclear factor-light-chain-enhancer of activated B (NF-kB) and its downstream targets cyclin D1 and cyclooxygenase-2 (COX2) in the MDA-MB-231 breast cancer cell line. This impact is thought to be connected to the fact that Ole reduces the expression of serine/threonine kinase (Akt) and IB [28], which are components of the NF-kB activation cascade (Figure 2) [29]. In vitro, Ole induced anti-proliferative effects on both TCAM-2 and SEM-1 cells by inhibiting the NF-κB pathway [30]. Ole was also reported to not affect IB in HT 29 colon cancer at concentrations of 400 and 800 µM [31]. The standard mechanism of NF-kB activation necessitates the phosphorylation of its inhibitor proteins, such as IB, but the non-canonical pathway does not [32].

The COX2 pathway is linked to colon cancer because it promotes proliferation and angiogenesis [33]. This is due to increased prostaglandin production. Ole was demonstrated to downregulate COX2. The suppression of the transcription factor cAMP response element-binding protein (CREB) is linked to this effect [16]. The downregulation of COX2 in colon cancer could be linked to the downregulation of the wnt/-catenin pathway by Ole [34]. Many malignancies, including gastric [35], colon [34], and endometrial [36], were linked to hyperactivation of the wnt/-catenin pathway. Because of the capacity of the wnt/-catenin pathway to activate COX2, studies on the use of non-steroidal anti-inflammatory drugs (NSAIDs) in cancer prevention and treatment have been conducted [37].

B-cell lymphoma 2 (Bcl2); NF-kB; the wnt/-catenin pathway [38]; and peroxisome proliferator-activated receptor (PPAR), which plays a key role in adipogenesis and is linked to obesity, insulin-dependent treatment [39], and osteoporosis [38], were discovered to modulate many gene expressions that promote adipogenesis but are also linked to cancer.

Because of its high expression in prostate, ovarian, cervical, follicular thyroid, esophageal, and testicular cancers, new studies looked at the role of PPAR in cancer [40]. According to one study, PPAR agonists may increase the risk of colorectal cancer [31]. High levels of PPAR expression have been associated with cell proliferation and tumor development inhibition [40], but it is yet unclear whether this is beneficial. It was discovered that HT, not Ole, is responsible for upregulating PPAR gene expression in HT-29 colon cancer cells in vitro [31].

Because several of these pathways are linked to the epithelial–mesenchymal transition [41], evidence of Ole’s ability in preventing osteoporosis has led to the hypothesis that Ole can also prevent bone cancer proliferation [6]. Ole can influence numerous pathways, including wnt/-catenin [41] and MMP and their inhibitors, as well as upstream activators of NF-kB [42] such as mitogen-activated protein kinases (p38 MAPK), extracellular signal-regulated kinase (ERK), and AKT [43]. Therefore, it is possible that Ole can prevent bone tumor formation by altering the tumor microenvironment [41]. The properties of the tumor microenvironment, or premetastatic niche, are significant aspects of cancer [44]. Many of those influenced by Ole, including VEGF, MMP, and NF-kB, are involved in this niche.

Ole (370 µM) possesses an anti-metastatic effect in MDA-MB-231 cells, which is thought to be attributable to the increased tissue suppression of metalloproteinase activity, which improves apoptosis and inhibits the activity of matrix metalloproteinase, (MMP) (promotes tumor cell metastasis) [5]. In glioma cells, the upregulation of MMP, which promotes invasion, was also observed. Ole (200/400 µM) considerably reduces the expressions of MMP-2 and MMP-9 in U251 and A172 hepatocarcinoma cells [45], while Ole (400 µM) dramatically reduces MMP-7 in HepG2 hepatocarcinoma cells [46]. MMP-2 plays a variety of roles in cancer angiogenesis, invasion, and tolerance [47]. MMP-7 and MMP-9, on their own or in combination, can induce tumor angiogenesis [48]. The mechanism by which MMP-9 and MMP-7 exert both angiogenic and pro-angiogenic effects is currently unknown, and more research is needed [48]. The activation of the NF-kB pathway has been shown to enhance MMP-9 production [27], and because Ole affects multiple components of the NF-kB pathway, the effects on MMP might be attributable to this [28,34].

In thyroid cancer cell lines, TPC-1 and BCPAP were used to confirm the antiproliferative activity of Ole and peracetylated Ole compared with non-tumor TAD-2 cell lines [49]. It was demonstrated that the antiproliferative activity was seen at concentrations below 100 M. The reduced phosphorylation of ERK and Akt [49], which are critical in cancer dissemination and invasion [50,51], was linked to this activity. Ole (100 and 500 µM) also inhibits LNCaP and DU145 prostate cell proliferation by causing necrosis [52].

AMPK (5′ adenosine monophosphate-activated protein kinase), CK1 or p21 are Cyclin-dependent kinase inhibitors, GF (growth factor), GRB2 (growth factor receptor-bound protein 2), MEK (mitogen-activated protein kinase), *MDM2* (murine double minute 2), RAF (rapidly accelerated fibrosarcoma), RTK (receptor tyrosine kinase), p53 (tumor protein p53), and p70S6K (70-kDa ribosomal protein S6 kinase). The figure highlights Oleuropein’s main signaling effects in reducing cancer cell proliferation and survival as well as its influence on these molecules as a chemotherapeutic drug that induces apoptosis by inhibiting the AKT signaling cascade and p53. Oleuropein raises the expression of the proapoptotic proteins p53 and Bax while decreasing the expression of the antiapoptotic proteins Bcl-2 and HIF-1. It also targets particular pathways that inhibit AKT [5].

Cannabinoid receptors (CB) are considered new anti-cancer targets because of their relevance to cancer progression and proliferation. In particular, CB1 is considered a tumor suppressor, and activation of this receptor initiates many cascades that prevent cancer onset, progression, and proliferation due to diverse mechanisms in multiple cancer cells, such as GI, lung, breast, and brain, prostate, pancreas, and thyroid [53]. The proliferative effect of lowering CB1 levels in cancer is inhibited by the upregulation of this receptor through Ole (50 µM) administration in human colon Caco-2 cells [54]. Ole (50 µM) also prevents the onset of colon cancer progression in vitro and in vivo through upregulation of the gene coding for CB1 [16]. In SH-SY5Y neuroblastoma cells, Ole (350 µM) significantly inhibits cell migration in vitro [55], and the mechanism behind this is still unknown.

**Table 1:** Anti-proliferative effects of Ole.

In Vivo/In Vitro	Type of Cancer/Cell	Anti-Proliferative Effects of Ole	References
MCF-7 in vitro	Breast cancer cells	- Enhances tissue inhibition of metalloproteinase activity - Enhances apoptosis - Reduces the activity of MMP (promotes metastasis of tumor cells)	[25]
MDA-MB-231 in vitro	Breast cancer cells	- Inhibits NF-kB and its downstream target cyclin D1 and COX2	[28]
TCAM-2 and SEM-1 in vitro	Seminoma Cells	- Inhibits the NF-kB pathway	[30]
HT 29 in vitro	Colon cancer cells	- Found to affect nuclear factor of kappa light polypeptide gene enhancer in B-cell inhibitor alpha (IκBα), which affects NF-kB canonical pathway - Downregulates COX2 and the wnt/β-catenin pathway - Inhibits the p38 pathway and transcription factor CREB - Alters PPARγ concentration	[31]
U251 in vitro	Glioblastoma cells	- Decreases the expressions of MMP-2 and MMP-9	[45]
A172 in vitro	Glioblastoma cells	- Decreases the expressions of MMP-2 and MMP-9	[45]
HepG2 in vitro	Hepatocarcinoma cells	- Decreases the expression of MMP-7	[46]
TPC-1 and BCPAP in vitro	Thyroid cancer cell	- Reduces phosphorylation of ERK and Akt	[49]
LNCaP and DU145 in vitro	Prostate cancer cell	- Induces necrotic cell death	[52]
Caco-2 cells in vitro	Human colon cell	- Upregulates the CB1 receptor	[54]
SH-SY5Y in vitro	Neuroblastoma cells	- Inhibits cell migration; unknown mechanism	[55]

### 3.2. Anti-Angiogenic and Apoptotic of Ole

Ole’s anti-angiogenic effect (Table 2) in in vivo breast cancer cell lines may be mediated by lowering the vascular endothelial growth factor (VEGF) at doses of 225 mg/kg/day for 3 weeks, administered in distilled water via gastric lavage [9]. This conclusion is supported by the fact that Ole (150 and 225 mg/kg/day), in vivo, decreases breast tumor volume, and this is related to the increase in endostatin expression [9].

In vivo and in vitro studies indicate that endostatin competes with VEGF to bind its receptor, which further prevents its phosphorylation and downstream pathway activation [9,56]. In the in vitro MDA-MB-321 breast cancer cell line, Ole induced cysteine proteases with aspartate specificity (caspase)-3 cleavage at a concentration of 200 µM, which resulted in the induction of cancer cell apoptosis [14]. The same was also observed in the in vitro NSCLC H1299 lung cancer cell line in which Ole, at the same concentration, caused a significant increase in cytochrome c and thus caspase-3 [57]. Ole’s effects on PARP cleavage might be modulated in part by caspase-3 cleavage at 100 µM concentration [28]. A study conducted to elucidate the effects of Ole on gene expression of breast cancer cells showed that in MIDA-MB-468 in vitro cells, Ole (250 µM in water) increased the expression of many caspases including caspase 1 and 14, which have a role in initiating apoptosis [58], while in MDA-MB-231 in vitro cells, Ole (500 µM in water) caused an increase in caspase 4 expression, which also promotes apoptosis [58].

Bcl-2-associated X protein (Bax) and Bcl2 have been demonstrated to promote apoptosis in U251 and A172 glioma cells by increasing caspase-3 and 9 expressions [45]. Bax and Bcl2 are proteins that regulate the apoptotic pathway in mitochondria through caspases. Bax is pro-apoptotic (promotes death), whereas Bcl2 is anti-apoptotic (prevents apoptosis and promotes survival).

Ole (200 µM) raised the ratio of Bax/Bcl2, favoring the apoptotic pathway in MIA PaCa-2 pancreatic cancer cells better than HT (100 µM), although this is not found in healthy cell lines [59]. Similar effects were observed in NSCLC H1299 lung cancer cells in vitro at 200 µM [57], breast neuroblastoma in vitro at 350 µM [55], and Hela cervical cancer cells in vitro at 200 µM [24]. Evidence suggests that Ole (200 µM) may affect the proapoptotic gene tumor protein P53 (p53) as well as the Bax/bcl2 ratio, which favors apoptosis. P53 and Bax are both proapoptotic, according to studies on MCF-7 in in vitro breast cancer cells [25]. Ole caused apoptosis in breast [25] and colon tumors at 400 and 800 µM [31] but not in U251 and A172 in in vitro glioma cancer cells at 200 or 400 µM [45]. Ole (200 µM) enhanced apoptosis in in vitro MIA PaCa-2 pancreatic cancer cells via the dimerization of c-Jun and c-Fos into API [59]. In addition to Bax/Bcl2, Ole (200 µM) promoted apoptosis in in vitro MIA PaCa-2 pancreatic cancer cells via the dimerization of c-Jun and c-Fos into AP1 [59]. In in vitro, Ole promoted cell apoptosis in TCAM-2 and SEM-1 cells by concomitantly enhancing the pro-apoptotic potential in these cells by the overexpression of BAX [30].

Another important mechanism in which Ole has a role in apoptosis is the p38 MAPK pathway [57]. One study found that Ole’s (200 µM) apoptotic ability is mediated by the p38/ATF-2 pathway in in vitro NSCLC H1299 lung cancer [57]. Similar results were seen in MCF-7 breast cancer [25], Hela cervical cancer cell [25], in vitro A549 lung cancer (200 µM) [60], and in vitro neuroblastoma SH-SY5Y [55] cell lines. However, treatment with Ole at doses of 200 or 400 in glioma cancer cells in vitro had no effect on the p38, ERK, or Jun N-terminal kinase (JNK) pathways [45].

In in vitro HT-29 human colon adenocarcinoma cells, Ole (400 and 800 µM) decreases the expression of the hypoxia-inducible factor-alpha (HIF-1α) protein [31]. HIF-1α plays a key role in the regulation of genes involved in cell motility, adhesion, and angiogenic cytokines [31]. In addition, Ole (0.04% of diet) was reported to reduce HIF-1α, and adipogenesis in B16F10 melanoma cells in mice fed a high-fat diet led in cancer progression prevention [61].

The Akt pathway is associated with apoptosis by activating the BCL2-associated agonist of cell death (Bad) and Forkhead box O [51]. Ole inhibits the Akt pathway in in vitro prostate cancer at 500 µM [52] and in vitro HepG2 hepatocellular at 60 µM cancers [62]. The ability of Ole (60 µM) to suppress phosphatidylinositol 3-kinase (PI3K)/Akt/NF-kB and phosphatidylinositol 3-kinase (PI4K)/Akt/mammalian target of rapamycin (mTOR) pathways was confirmed in in vitro HepG2 cells [62]. The Akt pathway is also suppressed by Ole (200/400 µM) in in vitro glioma cells, which are accompanied by regulating Bax, Bcl2, MMP-2, and MMP-9, which favor the apoptosis pathway [45].

Jnk’s role in cancer is confounded by its ability to act as both pro-and anti-apoptotic, depending on the cell type and what is activated or inhibited. The Jnk pathway promotes apoptosis in the absence of active NF-kB, according to studies. Although the significance of Jnk activation in the survival and proliferation of a variety of cancer cells is well understood, prolonged Jnk activation is still thought to induce apoptosis [63]. In in vitro HeLa cervical carcinoma cells, Ole (200 µM and dissolved in dimethyl sulfoxide) produced phosphorylation and activation of the Jnk pathway, and Ole-induced apoptosis led the mitochondria to upregulate Bax and cytochrome c [64]. The treatment of Ole (150 µM) increased the expression of pro-apoptotic Bcl2 in in vitro NSCLC A549 [65]. The findings also revealed that glyoxalase 2 (Glo2) expression may have a function in cancer and that Ole (150 µM) boosted Glo2 expression without changing its activity, implying a non-enzymatic effect [65]. The Glo2 enzymes may play a role in cancer by changing GSH metabolism [66]. All cells have this enzyme in their cytosol and mitochondria [66]. Glo2 has been shown to be regulated by P53 [66], while Ole has been proven to inhibit Akt pathway expression, which could affect P53 [65]. Furthermore, the study found that Ole promotes O2 depletion, which causes Akt activity to decrease, resulting in Glo2 upregulation [65]. The enhanced expression of mitochondrial Glo2 may be linked to the actions of Ole (150 µM) on the mitochondrial apoptotic pathway [65].

**Table 2:** Anti-angiogenic and apoptotic of Ole.

In Vivo/In Vitro	Type of Cancer/Cell	Anti-Angiogenic and Apoptotic of Ole	References
In vivo	Breast cancer cells	- Lowering vascular endothelial growth factor (VEGF)	[9]
MDA-MB-321 in vitro	Breast cancer cells	- Inducing cysteine proteases with aspartate specificity (caspase)-3 cleavage	[14]
NSCLC H1299 in vitro	Lung cancer cells	- Causing a significant increase in cytochrome c and thus caspase-3	[57]
MIDA-MB-468 in vitro	Breast cancer cells	- Increasing the expression of many caspases including caspase 1 and 14	[58]
MDA-MB-231 in vitro	Breast cancer cells	- Increasing caspase 4 expression	[58]
MIA PaCa-2 in vitro	Pancreatic cancer cells	- Raising the ratio of Bax/Bcl2	[59]
TCAM-2 and SEM-1 in vitro	Seminoma Cells	- Overexpression of BAX	[30]
NSCLC H1299 in vitro	Lung cancer cells	- Raising the ratio of Bax/Bcl2	[57]
In vitro	Breast neuroblastoma	- Raising the ratio of Bax/Bcl2	[55]
In vitro	Hela cervical cancer cells	- Raising the ratio of Bax/Bcl2	[24]
MCF-7 in vitro	Breast cancer cells	- Affecting the proapoptotic gene tumor protein P53 (p53) - Raising Bax/Bcl2 ratio	[25]
MIA PaCa-2 in vitro	Pancreatic cancer cells	- Dimerizing of c-Jun and c-Fos into API	[59]
NSCLC H1299 in vitro	Lung cancer cells	- p38/ATF-2 pathway	[57]
MCF-7 in vitro	Breast cancer cells	- p38/ATF-2 pathway	[25]
In vitro	Hela cervical cancer cell	- p38/ATF-2 pathway	[25]
A549 in vitro	Lung cancer	- p38/ATF-2 pathway	[60]
SH-SY5Y in vitro	Neuroblastoma	- p38/ATF-2 pathway	[55]
HT-29 in vitro	Human colon adenocarcinoma cells	- Causing down-regulation of hypoxia-inducible factor-alpha (HIF-1α)	[31]
B16F10 in vivo	Melanoma cells	- Reducing HIF-1α, and adipogenesis	[61]
In vitro	Prostate cancer	- Inhibiting Akt pathway	[52]
HepG2 in vitro	Hepatocellular	- Inhibiting Akt pathway - Suppressing phosphatidylinositol 3-kinase (PI3K)/Akt/NF-kB and phosphatidylinositol 3-kinase (PI4K)/Akt/mammalian target of rapamycin (mTOR) pathway	[62]
In vitro	HeLa cervical carcinoma cells	- Producing phosphorylation and activation of the Jnk pathway	[64]
NSCLC A549 in vitro	Non-small-cell lung cancer	- Increasing the expression of pro-apoptotic Bcl2	[65]

### 3.3. Antioxidant Properties of Ole

Ole promotes cell damage and functions as a pro-oxidant, which contributes to cell death, according to studies on in vitro MCF-7 breast cancer cells [25]. This impact is apparent at IC50 and 2× IC50 for 48 h and at all tested doses for 72 h. Ole’s antioxidant properties stem from its capacity to chelate metal ions such as copper and iron, which catalyze free radical production reactions, as well as its ability to inhibit several inflammatory enzymes [67]. The researchers also revealed that greater copper levels and copper complexing with Ole are responsible for the effects of Ole on SH-SY5Y neuroblastoma cancer cells in vitro. According to the study, copper is responsible for Ole’s ability to kill cancerous tissue [68]. Copper is a vital nutrient in the body, acting as a cofactor in a wide range of enzymatic operations as well as a structural component of proteins. Copper dysregulation or increase has been associated with lymphoma; reticulum cell sarcoma; bronchogenic and laryngeal squamous cell carcinomas; and cervical, breast, stomach, and lung cancers [69].

**Figure 3:**
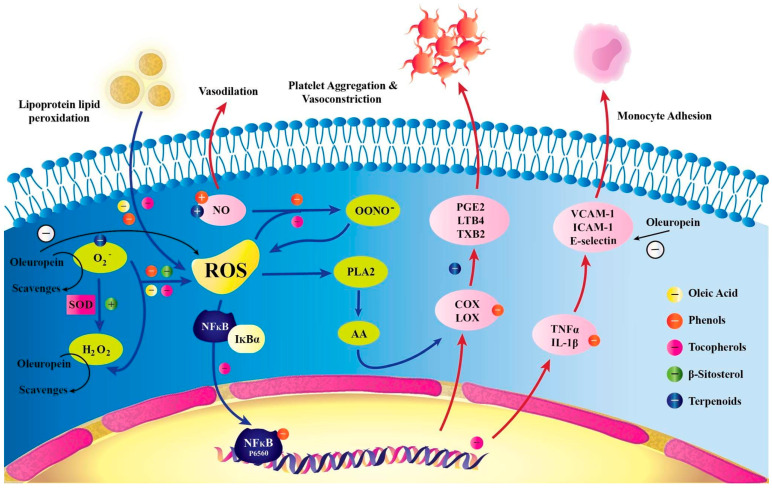
Proposed antioxidant mechanism of Ole. ICAM-1 (intercellular adhesion molecule 1), LTB4 (lipoxygenase), NO (nitric oxide), ONOO^−^ (*peroxynitrite*), PGE2 (prostaglandin E_2_), *TXB2* (thromboxane B2), and VCAM-1 (vascular cell adhesion molecule 1). Oleic acid, oleuropein, and beta-sitosterol are the three major components of olive oil that reduce intracellular ROS. O2 levels may drop as a result of -sitosterol and terpenoid oleanolic acid. Tocopherols and phenolics, which assist lower lipid peroxidation and scavenge intracellular ROS and free NO-, also inhibit the formation of OONO-. NFjB is activated by ROS to increase gene expression, and -tocopheryl succinate inhibits this mobilization. The level of molecule and eicosanoid adhesion is affected by NFjB’s modulation of LOX and COX expression. To protect the endothelium from vasoconstriction, platelet aggregation, and monocyte adhesion, phenolics, triterpenoids, and tocopherols reduce LOX and COX activities whereas IL-1b expression is inhibited by phenolics and tocopherols. The increase in NO caused by oleeuropein and oleanolic acid has vasodilatory effects [72].

The antioxidant effects of Ole and its derivatives are mediated by the breakdown of the radical chain [67], as shown in Figure 3. Ole (100 µmol/L dissolved in dimethyl sulfoxide) showed partial antioxidant and pro-oxidant characteristics in HepG2 hepatocarcinoma cells in vitro, with no harmful effects, according to one study. The activation of reactive oxygen species (ROS) is responsible for this pro-oxidant property [15]. Furthermore, the antioxidant impact of Ole is dependent on the cell type, time, and concentration of exposure [15,59]. Antioxidant effects were identified in non-malignant tissues; however, pro-oxidant stimulating ROS activation was seen in cancer tissue such as breast, prostate, and leukemia HL-60 cells [15]. Furthermore, a concentration larger than 100 µmol/l caused cell death, whereas a concentration less than that had an antioxidant effect [15]. In vitro, Ole (500 µM) possesses antioxidant and pro-oxidant effects on non-tumorous BPH-1 prostate cells as well as LNCaP and DU145 tumor cells [52]. Furthermore, an increase in the heme-oxygenase 1 (HO-1) enzyme at doses of 100 and 500 µM, which is a potent antioxidant containing thiol groups, is thought to be the mechanism by which the antioxidant action is exclusive to BPH-1 cells [52]. Several antioxidants, including glutathione (GSH) and others, need the presence of thiol groups [70]. Furthermore, rather than a change in GSH production via glutamyl cysteine synthetase (GCS) expression, the antioxidant impact in the in vitro prostate DU145 cell line is likely to be associated with a reduction in ROS [52]. According to certain studies, Ole’s antioxidant action has a chemo-protective effect, as evidenced by the fact that it slows colon cancer progression [16]. In in vitro TPC-1 and BCPAP thyroid cancer cell lines, the antioxidant effect of Ole and peracetylated Ole at concentrations of 100 µM was also detected [49]. Ole (as a dissolved solution in culture conditions) is thought to minimize oxidative stress by modulating intracellular GSH, a powerful antioxidant molecule. This was seen in vitro in human glioblastoma cells (U87) by Ole at 10 µM. Furthermore, Ole pre-treatment dramatically reduced NO and inducible nitric oxide synthase iNOS gene expression in these cells [71], supporting the chemo-protective role. The capacity of ROS to activate the Akt pathway could explain why giving Ole to cancer cells causes cell cycle arrest. Because ROS plays such an important part in cancer [72], it may potentially play a function in malignancy. Ole promotes ROS production in cancerous tissues while also inhibiting the Akt pathway, as previously mentioned. This dual impact could lead to an increase in intracellular ROS, which could lead to cell arrest [7,72] (Table 3).

### 3.4. Ole and Cell Viability

Breast cancer is divided into three subtypes based on chemotherapeutic sensitivity: (1) estrogen receptor-positive (ER+); (2) overexpressing human epidermal growth factor receptor 2 (HER2+), which can be ER+ or ER− and (3) triple-negative (TN), which lacks estrogen, progesterone, and HER2 receptor expression [5]. In HER2+ breast cancer cells, Ole aglycone (6.25–100 µM) was observed to reduce cell viability and apoptosis. Reduced extracellular domain cleavage, autophosphorylation, and HER2 expression are all linked to this action [5]. Ole failed to diminish cell viability in vitro hepatic cancer cells [15], but it did reduce cell viability in pancreatic cancer cells [59]. Furthermore, this effect was found to be specific to tumor cells and not to healthy cells [59]. A study comparing the effects of Ole on ER-negative MDA-MB-231 and ER-positive MCF-7 in in vitro cells found that the former is more responsive to Ole treatment than the latter at concentrations of 100 µM [28]. Most recently, a study carried out by Bossio et al. demonstrated that Ole (15–200 μM) has an inhibition effect on the cell viability assay in a dose-dependent manner in both intra- and extragonadal TCAM-2 and SEM-1 seminoma cells [30].

### 3.5. Ole and Cell Cycle Arrest

**Figure 4:**
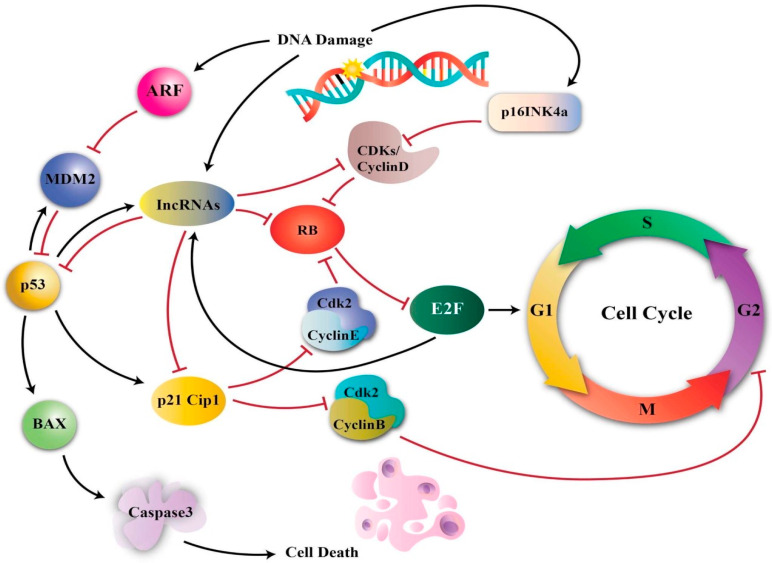
An illustration of the effects of p53 and p21 on the cell cycle. ARF (adenosine diphosphate-ribosylation factor) and lncRNA (long non-coding RNAs). P161NK4a and p14ARF modulate the activities of RB and p53. RB promotes cell cycle arrest in G1 and regulates entry into S phase by inhibiting E2Fs. P53 mediates apoptosis and G1 and G2 arrest. Additionally, p53 function reduction causes chromosomal instability. Different IncRNAs affect the expression of cyclins, including CDKs, CKIs, Prb, E2F, and P53, to govern the cell cycle. Additionally, some IncRNAs are produced as a result of DNA damage and stop the advancement of the cell cycle [73].

The effects of Ole on the cell cycle have been verified in numerous research. Ole (100 µg/mL) was shown to be more effective than its derivative HT (25 µg/mL) in slowing the transition from G1 to S phase in MCF-7 breast cancer cell lines [5,26]. Ole also produced a delay in the S phase cell cycle in MDA-MB-231 breast cancer cells in another in vitro investigation by upregulating p21 [14,73,74] (Figure 4). The effect of Ole on the MDA-MB-231 cell cycle was shown to be at the sub-G1 phase [54].

Ole also slowed mitosis, which is thought to be related to a reduction in COX-2, which interrupts the cell cycle in the G2/M phase. This effect was detected in mice given 150 and 225 mg/kg/day of Ole diluted in distilled water by gastric lavage [9]. The down-regulation of cyclin-D1, 2 and 3, and CDK4 and 6 gene expression, as well as the up-regulation of p53 [59] and cyclin-dependent kinase inhibitor gene expression are likely to be responsible for the cell cycle arrest effects of Ole (350 µM) on in vitro neuroblastoma [55]. In neuroblastoma, Ole has a particular effect on the G1/S phase [58]. Cyclins and their allosteric activators, cyclin-dependent kinases (CDK), play critical roles in cell cycle control. The overexpression of cyclins, particularly cyclin D1, has been confirmed to have a role in cancer. Cyclin D1 can regulate the G1/S phase of the cell cycle via retinoblastoma (Rb) phosphorylation. Pancreatic cancer; non-small cell lung carcinoma; breast, NSCLC, head, and neck squamous cell carcinoma; melanoma; and endometrial, colorectal, and mantle cell lymphoma malignancies all have hyperphosphorylation of Rb [73].

Furthermore, Ole (200 µM) or its metabolite HT (100 µM) suppresses the cell cycle in the G2 phase in MIA PaCA-2 pancreatic cancer cells in vitro [59].

Ole was discovered to promote the concentration of cells in the G2/M phase, inhibiting the growth of H1299 lung cancer at 200 µM [57] and HeLa cervical cancer lines at 200 µM [64]. The cell cycle arrest phase is dependent on Ole concentration; for example, at a lower concentration (50 µM), the cell cycle may be arrested in the S phase rather than the G phase [64]. Ole’s capacity to affect the cell cycle could be attributed to its ability to alter p53 expression, which leads to cell cycle arrest as well as its effects on p21, a CDK inhibitor [64]. Furthermore, since Ole increases the Jnk pathway, p53 and p21 expression is elevated, as Jnk activation influences p53 and p21 (Figure 4) [64,75].

### 3.6. Ole as a Cytoskeleton Disruptor

Ole disrupted actin filaments in the cytoskeleton of breast cancer cells (MCF-7) within two hours in vivo. Interestingly, when Ole was combined with D-glucose, the potential of Ole to disrupt the cytoskeleton was reduced, suggesting the involvement of the glucose transporters (GLUTs) [76]. Ole’s capacity to cause cell rounding in ovarian cancer cells is attributed to the disruption of the actin cytoskeleton, which hinders these cells from replicating and invasiveness [14].

### 3.7. Ole and Fatty Acid Synthase

**Figure 5:**
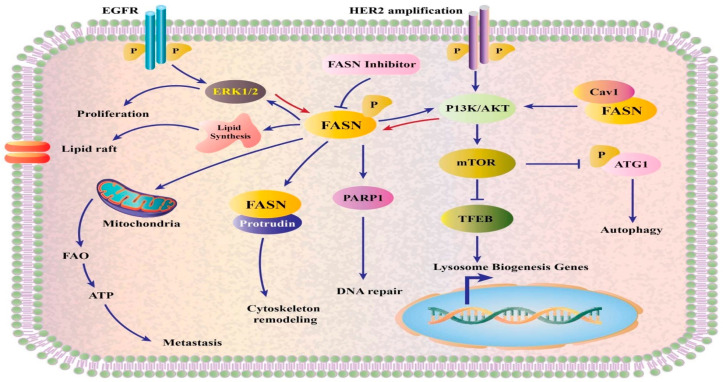
Proposed mechanism of FASN in cancer [78]. Atg1 (autophagy-related 1), CAV1 (caveolin-1), FAO (fatty acid oxidation), and TFEB (transcription factor EB). Expression and activity of FASN are impacted by EGFR and HER2. Lipid synthesis, PI3K/AKT pathway signaling, extracellular regulated kinase (ERK)1/2 signaling, and transcription of genes involved in lysosome biogenesis are all mediated by FASN. Proliferation is accelerated by FASN overexpression.

Ole’s anti-cancer benefits could be attributed to its capacity to inhibit the fatty acid synthase enzyme (FASN), which could be linked to the fact that Ole modifies gene expression and enzymatic activity of this enzyme, as seen in colon cancer cell lines SW620 and HT-29 in vitro [16]. FASN is overexpressed in a variety of cancers, including prostate, ovarian, breast, endometrial, thyroid, colorectal, bladder, lung, thyroid, oral, tongue, esophageal, hepatocellular, pancreatic, and gastric carcinomas; malignant melanoma; mesothelioma; nephroblastoma; and retinoblastoma; as well as soft tissue sarcoma [77]. In tumor cells, the overexpression of this enzyme impacts mitochondrial activity as well as peroxisomes, nuclei, and endoplasmic reticula. Furthermore, unlike healthy cells, cancers use fatty acid in de novo synthesis regardless of circulating lipid levels [78]. As illustrated in Figure 5, FASN has an impact on a variety of pathways, including the AKT and ERK1/2 pathways.

### 3.8. Ole and Inflammation

Inflammation is a condition that occurs in injured cells and is characterized by the production of pro-inflammatory cytokines such as IL-6 and IL-1, as well as the activation of COX and iNOS [1]. The activation of the NF-kB pathway by these cytokines, as well as the overexpression of MMP are all part of the inflammation process [1]. There is a well-established relationship between chronic inflammation and cancer progression and development. At various phases of cancer progression, all immune cells are implicated. This persistent inflammation as well as the proinflammatory cytokines IL-1 and IL-6 promote tumor formation in lung and breast cancer models [42]. Many proinflammatory cytokines, including IL-6, IL-8, IL-1, and TNF-, have been identified as altering bone tumor angiogenesis, progression, and tumor microenvironment [41]. One of the most important properties of a premetastatic niche is inflammation [45]. Ole aglycone (10 µM for 5 weeks) suppresses the establishment of a premetastatic niche by lowering the release of IL-8 and MMP in senescence-associated secretory-phenotype cells [1].

In vitro (human whole blood) investigations have found that Ole (104 µM) reduces IL-1 relative to the other olive oil phenols and induces a decrease in the expressions of IL-6, iNOS, NF-kB, and JNK in murine RAW 264.7 cells at 300 µM [1], while the effects of Ole on colonic biopsies taken from human patients with ulcerative colitis showed that Ole decreases COX and IL-17, which are upregulated in this disease. This may be questionable because the metabolism of Ole in these models may be different than in humans, as stated in the Metabolism of Ole section. However, the effects of Ole on colonic biopsies taken from human patients with ulcerative colitis showed that Ole decreases COX and IL-17, which are upregulated in this disease [1].

The only olive phenol that has anti-inflammatory properties in polymorphonuclear cells isolated from human samples was Ole (80 µg/mL) [79]. TNF secretion is reduced, and this impact is apparent at greater concentrations (320 µg/mL) [79].

## 4. Derivatives of Ole

Due to Ole’s hydrophilic character, researchers found that more stable hydrophobic derivatives of Ole had stronger anti-proliferative and antioxidant effects in MCF-7 and T-47D breast cancer cells in vitro [67]. The improved efficacy of the derivatives, according to the researchers, is related to the creation of better antioxidants by enzymatic breakdown of the derivatives; additionally, the compounds are more hydrophobic, which facilitates membrane integration [67]. A recent study found that liposomes containing Ole and HT are an optimal delivery strategy for delivering Ole directly into cells without the complications and variables associated with Ole metabolism [80]. The antioxidant activity of the more lipophilic Ole acetylated derivatives, particularly acetylated Ole aglycone, was found to be higher in mice given Ole, Ole aglycone, and their semisynthetic acetylated derivatives [81]. In another study, the acetylation of Ole’s glucose moiety increased lipophilicity and permeability as well as improved anti-inflammatory and antioxidant profiles in murine peritoneal macrophages [80].

Many researchers conducted trials employing different drug delivery technologies to better distribute Ole inside infected cells since Ole has a variable metabolism and bioavailability profile. Nanostructured lipid carriers loaded with Ole were used in one of these studies. These carriers showed good encapsulation efficiency and effective Ole loading. Ole showed sustained release kinetics after assessing the features of the delivery system, indicating that Ole was stable inside the lipid carriers and did not experience any degradation, which is further verified by antioxidation activity against A549 lung cancer cells [81].

## 5. Ole in Combination with Chemotherapy

A combination of Ole with other anticancer agents may be beneficial in chemotherapeutic resistance. This was observed from the in vitro and in vivo experiments of the combinations of SKBR3/Tzb100 in SKBR3/Tzb100 (trastuzumab with Ole aglycone) [5,82] and doxorubicin with Ole, [5,14]. In in vivo models of breast cancer mice, the combination of Ole (50 mg/kg) and doxorubicin (1.5 mg/kg) decreased tumor growth and size, caused apoptosis, and affected the protein cytoskeleton of tumor cells [14,82]. This combination also suppressed Bcl2 and survival expression, both of which prevent apoptosis [14]. It also downregulated NF-kB, cyclin D1, and COX2; enhanced capsapse-3 cleavage and Bax expression; and boosted capsapse-3 cleavage and Bax expression. In in vivo male Winstar rats, Ole showed a protective effect when combined with cyclophosphamide [82].

In the case of cisplatin-induced renal impairment, combining Ole with cisplatin treatment could be beneficial. The ERK pathway has been shown to mediate Ole’s reno-protective action [83]. In vivo and in vitro investigations verified that ERK activation is linked to inflammation and apoptosis, as evidenced by the activation of downstream targets such as COX and TNFa [83]. In contrast to Ole monotherapy, adding Ole to 50 µM cisplatin significantly reduces NO levels and cell survival rate while also inducing cytotoxicity in in vitro HepG2 hepatocarcinoma cells [46]. Furthermore, Ole (100–400 µM) improved the efficacy of cisplatin (50 µM) to inhibit MMP-7 gene expression [40]. The same protective effect was observed in the lungs, stomach, and pancreas [82]. In in vitro human melanoma cells (A375), Ole was found to have a synergistic impact when combined with Decarbonize and Everolimus, as well as Vemurafenib [82]. When Ole (100 µM) was combined with 2-metoxyestradiol (10 µM) in human osteosarcoma cells, a synergistic effect was seen [82].

## 6. Summary and Conclusions

Ole’s anticancer properties are numerous and encompass numerous mechanisms that are presently being explored. Ole inhibits cell proliferation by interacting with the AKT/NF-kB pathway and COX2, PPAR, MMP, and CB receptors. Ole’s potential to impact PARPs, the Bax/Bcl2 ratio, the P38 MAPK pathway, HIF-1α, and the Akt and Jnk pathways disrupts cancer’s anti-apoptotic and angiogenic capabilities. Ole decreases cell viability, causes cell cycle arrest, and functions as a cytoskeleton disruptor in many cancer types in addition to its antioxidant properties and unusual complex formation with copper.

As concluded from various studies, Ole has a variable metabolism and bioavailability profile and it undergoes extensive first-pass metabolism. Ole metabolism is mediated by several metabolic processes, including de-glycosylation, hydrolysis, oxygenation, and methylation. More human studies are needed to discover the various factors influencing Ole metabolism and absorption. Derivatives of Ole have improved its efficacy for example as an antioxidant and for anti-proliferation.

Ole have aided in cancer treatment over the years. Ole functions as an anti-cancer agent by having anti-proliferative, anti-angiogenic and apoptotic, antioxidant, fatty acid synthase inhibiting, cytoskeleton disrupting, and anti-inflammatory properties. Oleuropein effects on cancer cells are influenced by Ole concentration, exposure time, and cancer cell type. Ole has strong synergistic effects when combined with anticancer medicines such as doxorubicin and other. Ole is a prospective anticancer candidate that could be utilized as a supplement to existing anticancer therapy guidelines or as a recurrence prevention therapy.

## Figures and Tables

**Table 3:** Antioxidant properties of Ole.

In Vivo/In Vitro	Type of Cancer/Cell	Antioxidant Properties of Ole	References
MCF-7 in vitro	Breast cancer cells	- Promoting cell damage and functions as a pro-oxidant	[25]
SH-SY5Y in vitro	Neuroblastoma cancer cells	- Copper complexing with Ole	[68]
HepG2 in vitro	Hepatocarcinoma cells	- Breakdown of the radical chain	[15]
BPH-1 in vitro	Prostate cells	- Increasing in heme-oxygenase 1 (HO-1) enzyme	[52]
DU145 in vitro	Prostate cells	- Reducing ROS	[52]
TPC-1 and BCPAP in vitro	Thyroid cancer cell	- Minimizing oxidative stress by modulating intracellular GSH	[49]
U87 in vitro	Human glioblastoma cells	- Reducing NO and inducible nitric oxide synthase iNOS gene expression	[71]

## Abbreviations

Akt	Serine/threonine kinase
AMPK	5′ adenosine monophosphate-activated protein kinase
ARF	Adenosine diphosphate-ribosylation factor
Atg1	Autophagy-related 1
Bad	BCL2-associated agonist of cell death
Bax	Bcl-2-associated X protein
Bcl2	B-cell lymphoma 2
Caspase	Cysteine proteases with aspartate specificity
CAV1	Caveolin-1
CB	Cannabinoid receptors
CREB	cAMP response element-binding protein
COX2	Cyclooxygenase-2
ERK	Extracellular signal-regulated kinase
ER+	Estrogen receptor-positive
FAO	Fatty acid oxidation
FASN	Fatty acid synthase enzyme
GCS	Glutamyl cysteine synthetase
GF	Growth factor
GRB2	Growth factor receptor-bound protein 2
Glo2	Glyoxalase 2
GLUTs	Glucose transporters
GSH	Glutathione
HER2+	Human epidermal growth factor receptor 2
HIF-1α	Hypoxia-inducible factor-alpha
HER2+	Human epidermal growth factor receptor 2
HT	Hydroxytyrosol
ICAM-1	Intercellular Adhesion Molecule 1
IκBα	Nuclear factor of kappa light polypeptide gene enhancer in B-cell inhibitor alpha
Inos	Inducible nitric oxide synthase
JNK	Jun N-terminal kinase
lncRNA	Long non-coding RNAs
LTB4	Lipoxygenase
MDM2	Murine double minute 2
MMP	Matrix metalloproteinase
MEK	Mitogen-activated protein kinase
mTOR	Mammalian target of rapamycin
NF-kB	Nuclear factor-light-chain-enhancer of activated B
NO	Nitric oxide
NSAIDs	Non-steroidal anti-inflammatory drugs
Ole	Oleuropein
ONOO−	Peroxynitrite
PARPs	Poly (ADP-ribose) polymerases
PGE2	Prostaglandin E2
PI3K	Phosphatidylinositol 3-kinase
PI4K	Phosphatidylinositol 4-kinase
PPAR	Peroxisome proliferator-activated receptors
P38 MAPK	Mitogen-activated protein kinases
P53	Tumor protein P53
p70S6K	70-kDa ribosomal protein S6 kinase
RAF	Rapidly accelerated fibrosarcoma
ROS	Reactive oxygen species
RTK	Receptor tyrosine kinase
TFEB	Transcription factor EB
TN	Triple-negative
TXB2	Thromboxane B2
VCAM-1	Vascular cell adhesion molecule 1
VEGF	Vascular endothelial growth factor
